# 

**Published:** 1901-07

**Authors:** 


### INDEX TO VOLUME XVII.

*Page*

Abstracts and Selections............16-18, 53-56, 96-98, 133-140, 172, 207-216, 256-262, 296-307, 341-346, 385-387

Accouchement Force .......................................... 375

American Congress of Tuberculosis............................ 432

American Medical Association.............................249, 434

A New Medical Society........................................ 168

A New Subscriber............................................. 22

Apomorphia—Its Origin and Therapeutics, as Applied in Asphyxia, Drowning, Lightning Stroke, Emetic in Ejecting Poisons from the Stomach, Labor, Fever, Strangulated Hernia................... 84

A School for Health Officers................................. 217

As Others See Us............................................. 430

Aspiration in Knee Joint Effusion After Traumatism............... 283

Association of Health Officers of Texas.......................... 8

A Suit for Damages........................................... 336

Austin District Medical Society................................ 168

Books and Magazines..........27-29, 64-70, 105-108, 150-154, 182-185, 226-229, 265-272, 309-313, 350-356, 390-398

Brazos Valley Medical Association........................249, 434

Camp Life for Consumptives.................................. 162

Camp Treatment of Consumptives.............................. 175

Car Sanitation in Texas...................................... 381

Coming This Way at Last...................................... 430

Common Nasal Conditions as the Cause of Neuralgia of the Terminal Branches of the Fifth Nerve................................ 323

Criminal Abortion ........................................... 79

Dr. Winfield Ayers' Experience with Mercurol in the Treatment of Syphilis ................................................... 284

Enteroptosis ................................................ 60

Four Cases of Appendicitis, Illustrating Four Types of the Disease... 86

Going on Eighteen............................................ 461

Gonorrhea in the Female...................................... 327

Health Officers' Association of Texas......................... 249

"Irregularities" in the Texas Quarantine Department........248, 289, 335

Page

Letter of Instructions to Medical Societies in Affiliation with State Medical Association of Texas.................................. 426
Leucorrhea and its Treatment.......................................... 331

Malarial Hemoglobinuria .......................................... 198

Naevi: Report of a Case and Treatment of Electrolysis.............. 329
News and Miscellany.........24-27, 61-62, 103-105, 146-150, 178-182, 223-226, 263-264, 307-309, 346-349, 387-389, 478-482

On Axis-Traction Forceps .......................................... 1
Oklahoma Territorial Medical Association.......................... 170

Pelvic Peritonitis .......................................... 125
Perforated Intestine Searcher.......................................... 131
Preliminary Announcement and Program State Medical Association of Texas .......................................... 403
Publisher's Notes.............30-40, 70-78, 108-116, 154-156, 185-194, 229-233, 272-282, 314-322, 358-362, 398-402, 436-444, 482

Quarantine in Relation to the Present Statute Regulating Same..... 50

Recollections of the Big Dallas Meeting.......................... 472
Reorganization: Its Effect Upon the Country Society.............. 292
Reply to a G.-U. Critic.......................................... 363
Report of Committee on Constitution and By-Laws Texas State Medical Association .......................................... 411

San Antonio's Invitation to the A. M. A.......................... 466
Society Notes ..........................................95, 478
South Texas Medical Association.......................................... 200
State Medical Association of Texas.................................. 462

Texas State Medical Association..............................251, 338
The Advertising Doctor.......................................... 174
The Age of First Menstruation in the United States................ 89
The American Congress of Tuberculosis.............................. 295
The Association of Army and Navy Medical Officers of the Confederate States ..................................168, 250, 338, 339
The Cancer Parasite.......................................... 23
The Central Texas Medical Association Meeting.................... 294
The Chicago Clinic .......................................... 294
The Dallas Meeting.......................................... 466
The Dallas Meeting of the State Medical Association of Texas........ 429
The Drug Habit: Its Treatment.......................................... 157
The Ear and Kidney in Mild Scarlet Fever.......................... 326
The East Texas Medico-Chirurgical Society........132, 169, 254, 384, 434
The El Paso Meeting.......................................... 173
The Epileptics .......................................... 59
The Great Fraud on the Doctors: Substitution...................... 99

*Page*
The Increasing Sterility of American Women.................... 91
The Latest .................................................. 336
The "Melancholy Days"....................................... 176
That Mosquito Fallacy ...................................... 221
The New State Health Officer................................ 177
The North Texas Medical Association......................... 168
The Only Pebble on the Beach................................ 23
The President's Case........................................ 141
The President's Wound....................................... 167
The Public Health........................................... 128
The Record of Yellow Fever in the United States for the Past Does Not Sustain the Mosquito Theory.......................... 235
The Roberts-Hawley Goat Lymph Compound....................... 41
The Registration of Deaths.................................. 336
The State Association of Health Officers' Meeting........... 294
The State Board of Medical Examiners........................ 19
The State Board of Medical Examiners........................ 57
The Systematic Doctor....................................... 117
The Texas Medical College................................... 20
The Texas State Medical Association......................... 101
The Texas State Medical Association Meeting................. 294
The Treatment of Pneumonia, with Special Reference to Creosote.... 195
The United States Health Service............................ 293

West Texas Medical Association.......................204, 253, 295, 384
What Shall We do with Our Consumptives...................... 370
What the American Medical Association Asks of the State Societies.. 340

Yellow Fever and the Mosquito............................... 245

